# Generation and validation of a versatile inducible multiplex CRISPRi system to examine bacterial regulation in the *Euprymna-Vibrio fischeri* symbiosis

**DOI:** 10.1007/s00203-025-04354-8

**Published:** 2025-05-17

**Authors:** Brian Lynn Pipes, Michele Kiyoko Nishiguchi

**Affiliations:** Department of Molecular and Cell Biology, University of California, Merced, CA 95343 USA

**Keywords:** CRISPRi, Symbiosis, Vibrio, Squid, dCas9

## Abstract

The *Vibrio fischeri*—*Euprymna scolopes* symbiosis has become a powerful animal—microbe model system to examine the genetic underpinnings of symbiont development and regulation. Although there has been a number of elegant bacterial genetic technologies developed to examine this symbiosis, there is still a need to develop more sophisticated methodologies to better understand complex regulatory pathways that lie within the association. Therefore, we have developed a suite of CRISPR interference (CRISPRi) vectors for inducible repression of specific *V. fischeri* genes associated with symbiotic competence. The suite utilizes both Tn7-integrating and shuttle vector plasmids that allow for inducible expression of CRISPRi dCas9 protein along with single-guide RNAs (sgRNA) modules. We validated this CRISPRi tool suite by targeting both exogenous (an introduced mRFP reporter) and endogenous genes (*luxC* in the bioluminescence producing *lux* operon, and f*lrA*, the major regulatory gene controlling flagella production). The suite includes shuttle vectors expressing both single and multiple sgRNAs complementary to the non-template strand of multiple targeted genetic loci, which were effective in inducible gene repression, with significant reductions in targeted gene expression levels. *V. fischeri* cells harboring a version of this system targeting the *luxC* gene and suppressing the production of luminescence were used to experimentally validate the hypothesis that continuous luminescence must be produced by the symbiont in order to maintain the symbiosis at time points longer than the known 24-h limit. This robust new CRISPRi genetic toolset has broad utility and will enhance the study of *V. fischeri* genes, bypassing the need for gene disruptions by standard techniques of allelic knockout-complementation-exchange and the ability to visualize symbiotic regulation in vivo.

**Supplementary Information:**

The online version contains supplementary material available at 10.1007/s00203-025-04354-8.

## Introduction

The bioluminescent marine bacterium *Vibrio fischeri* is a widely adopted model organism for studies of symbiosis (with its host squid *Euprymna scolopes*) (Stabb and Visick 2014), biofilm formation (Yildiz and Visick 2009; Visick 2009), bioluminescence (Septer and Stabb 2012), and quorum sensing (Verma and Miyashiro 2013). Research in these fields has been increasingly facilitated by the development of progressively more sophisticated genetic tools to manipulate the *V. fischeri* genome. Genetic modification tools were first adapted for use in *V. fischeri* in 1992 with the demonstration of homologous insertional mutagenesis from conjugatable plasmid vectors (Dunlap and Kuo 1992). Motility was identified as the first *V. fischeri* phenotypic characteristic required for successful host colonization using a Mu dI 1681 transposon plasmid vector for random mutagenesis in 1994 (Graf et al. 1994). Tools for Tn7 transposon targeted insertional mutagenesis in *V. fischeri* were first demonstrated in 1997 (Visick and Ruby 1997). Development of both stable and suicide plasmid vectors (Dunn et al. 2006) further enhanced the ability to probe *V. fischeri* genetics. Subsequently, natural transformation based genetic modification tools using the forced over-expression of the natural transformation master regulator *tfoX* were adapted for use in homologous recombination based insertion/deletion/allelic exchange methods in *V. fischeri* (Pollack-Berti, et al. 2010). Further work enhanced the utility (Visick and Ruby 1997)*,* and transformation efficiencies (Fidopiastis et al. 2024) of induced natural transformation.

Methods to probe gene function through the controlled modulation of expression of a targeted gene were also developing and being adapted for use in bacteria, including *V. fischeri*. RNA interference methods, using plasmids vectors with inducible promoter expression of antisense RNA that is complementary to the mRNA of a targeted gene. This has been shown to repress gene expression, from mild to knock-out levels (Magistro et al. 2018), and also target multiple genes (Nakashima et al. 2012). Another use of inducible promoters to modulate gene expression in a titratable and reversable manner involved a Tn5 transposon genetic tool that was adapted for use in *V. fischeri* and used in the construction of strains in which IPTG-inducible promoters were randomly inserted into the genome to identify and control the expression of genes controlling phenotypic characteristics (Ondrey and Visick 2014).

While inducible control of gene expression by promoter replacement is a valuable tool to link gene to phenotype (Judson and Mekalanos 2000; Ondrey and Visick 2014) endogenous promoter replacement with non-endogenous inducible promoters may not allow for the normal range of expression of the inducible target gene. An alternative approach which retains the normal range of endogenous gene expression is CRISPRi (clustered regularly interspaced short palindromic repeats interference) repression of native gene expression (Qi et al. 2013). In CRISPRi, a mutated de-active (no nuclease function) form of the Cas9 protein (dCas9), in conjunction with a programable sgRNA guide, induces steric hindrance of RNA polymerase promoter binding and transcript expression leading to repressed protein expression (Qi et al. 2013). By using an inducible promoter, dCas9 promoted gene repression can be reversibly induced and relieved to normal physiologic levels in a temporal and spatial manner (Banta et al. 2020a, b). The sgRNA will usually target the promoter or 5’-end of a transcribed gene via complementary binding of the spacer sequence (20-nt for the commonly used SpdCas9 (from *Streptococcus pyogenes*) (Geyman et al. 2024; Qi et al. 2013). A constraint that limits the targetability of CRISPRi systems is that the targeted sequence must be located next to a protospacer adjacent motif (PAM) sequence (5′-NGG-3′ for *S. pyogenes* dcas9) (Didovyk et al. 2016), which means that true single base pair targeting of a given genetic loci is not yet possible, though the hunt is on for new PAM-less cas9 variants (Collias and Beisel 2021). Other limitations of CRISPRi include dcas9 toxicity that has been observed in some bacterial species at high dcas9 expression levels (Cui et al. 2018), and the possibility of off-target effects, where the dcas9-sgRNA complex binds to unintended DNA sequences that share partial homology to the intended target (Feng et al. 2021). Strategies to mitigate off-target effects include off-target detection, with several in silico tools, such as CRISPy-web (CRISPy-web), CRISPR Design Tool (synthego.com/products/bioinformatics/crispr-design-tool), and FlashFry (Naeem et al. 2020), which can identify gene specific potential PAM-adjacent 20-nt targeting sequences while also scanning the bacterial genome for potential off-target sequences with high homology. Locations of potential targeting sequences are ranked by the number of potential off-target sites found and their degree of homology (Naeem et al. 2020). It has been observed that when CRISPRi is targeted to a gene within an operon (a group of genes that are transcribed together as a polycistronic transcript), downstream genes in the operon are also repressed (Peters et al. 2016; Qi et al. 2013). This polar effect can make it challenging to accurately identify the function of individual genes within an operon. However, it can also be an advantage, as operons often contain genes involved in the same biological pathway, so silencing one gene can lead to the silencing of related genes.

The use of bacterial CRISPRi has been expanding, with applications ranging from the elucidation of gene functions and the screening of essential genes (Todor et al. 2021), to diverse projects in synthetic biology and metabolic engineering (Didovyk et al. 2016). CRISPRi systems have been adapted for use in diverse bacterial species, (Peters et al. 2019), (Banta et al. 2020a, b; Enright et al. 2024; Geyman et al. 2024), including members of the *Vibrionaceae* such as *Vibrio cholera* (Caro et al. 2019), *Vibrio natriegens* (Lee et al. 2019), *Vibrio casei* (Peters et al. 2019), and *Vibrio parahemolyticus* (Jiang et al. 2024). Recently the efficacy of the Mobile-CRISPRi Tn7 vector (Peters et al. 2019) for the targeted IPTG-inducible repression of a chromosomally integrated GFP reporter gene as well as the essential gene *rpoB* was demonstrated in *V. fischeri* (Geyman et al. 2024). When induced in culture, the chromosomally integrated dcas9/sgRNA Mobile-CRISPRi system significantly reduced reporter GFP expression levels and introduced a significant growth lag, demonstrating the utility of this system for targeted gene repression in *V. fischeri.*

In this work we have extended the utility of an inducible CRISPRi system in *V. fischeri* by incorporating a plasmid vector containing a multiplexed sgRNA expression cassette with three independently targetable sgRNA moieties within a nonrepetitive extra-long sgRNA array (Reis et al. 2019) for use with the Tn7 integrating IPTG-inducible (PLlac0-1 promoter) dcas9 expression plasmid vector pJMP1189, one of the Mobile-CRISPRi suite of vectors (Peters et al. 2019). Our *V. fischeri* CRISPRi system demonstrates inducible, titratable, and reversible repression of the expression of single or multiple targeted *V. fischeri* gene(s) in culture. The design, generation, and functional testing of our *V. fischeri* CRISPRi system in vitro, and its application in experiments probing the temporal monitoring of bioluminescence during the squid-*Vibrio* symbiosis establishment are described below.

## Results

### Functional testing of CRISPRi repression of mRFP gene expression

**Fig. 1:**
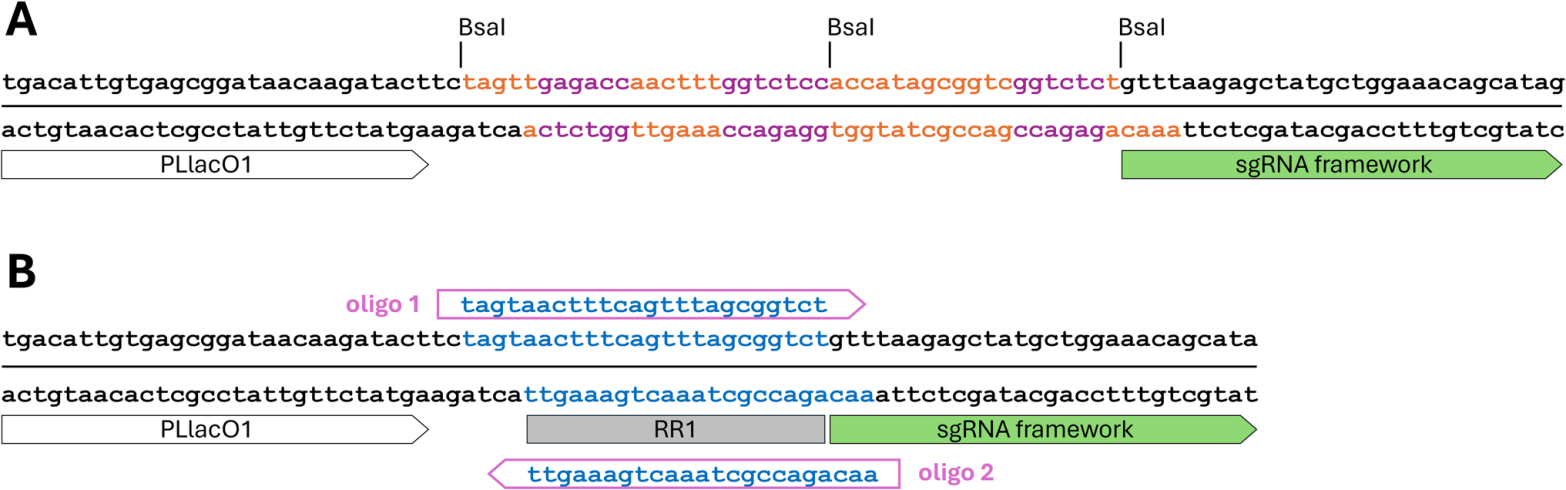
SgRNA spacer cloning. **A** the triple *BsaI* TIIS cloning site allows for efficient cloning of 20-nt spacer sequences in-frame with the adjacent sgRNA framework sequence. **B** Ligation of annealed 20-nt targeting oligos into the resulting empty BsaI-cut cloning site. The spacer shown is the (RR1) spacer used in this study to target the mRFP gene

**Fig. 2:**
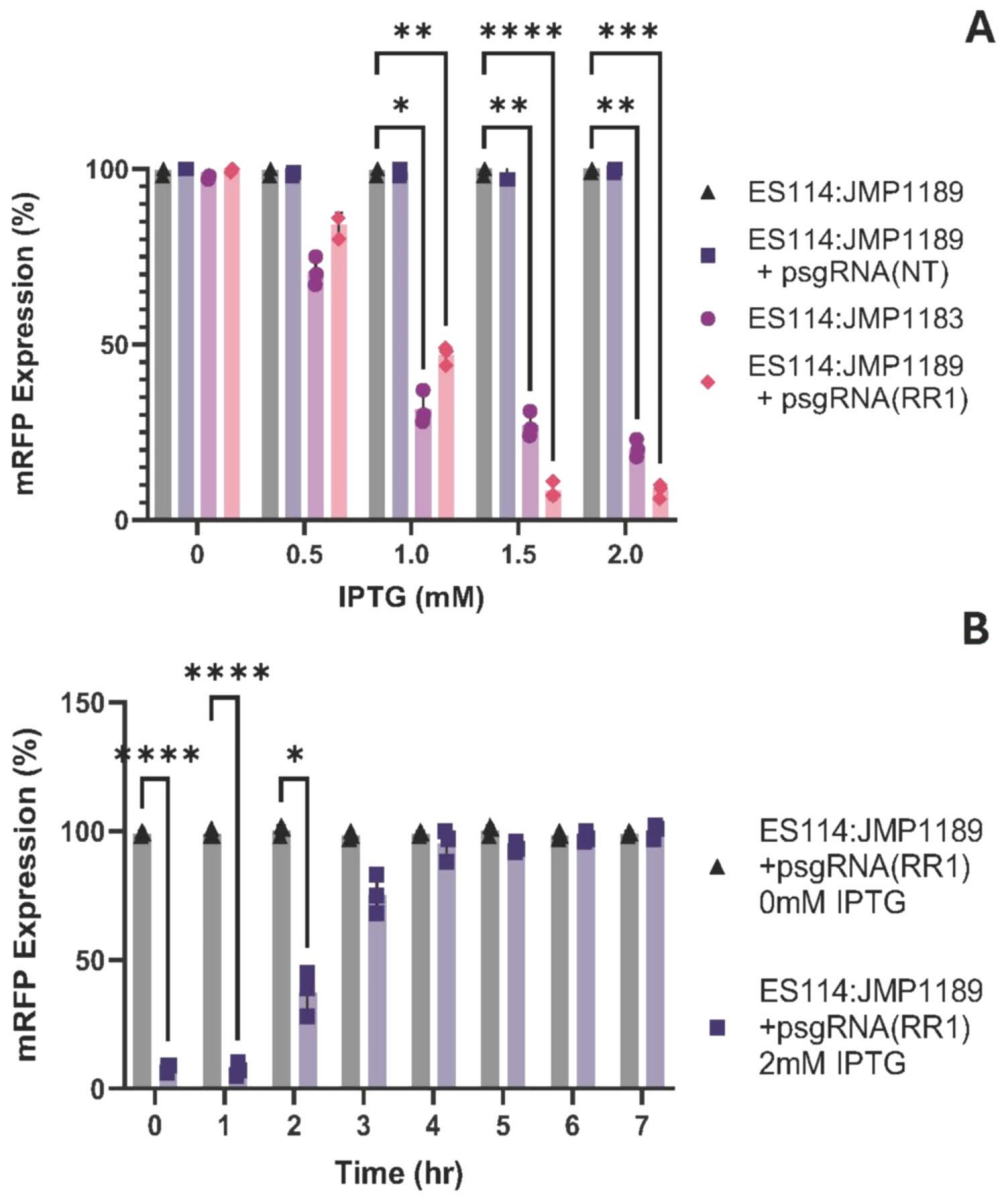
**A** Titratable repression of mRFP. mRFP expression was calculated as specific fluorescence (relative fluorescence units/OD_600_) of induced strains normalized to the control strain ES114:JMP1189. Values reported reflect the mRFP repression at mid-log growth (OD_600_ = 0.4). All values reported are mean values and error bars reflect the standard deviation from the mean. Statistics were done using a two-way ANOVA, with Šídák’s multiple comparisons test *: p < 0.05; ** p < 0.01; *** p < 0.001; **** p < 0.0001. **B** Reversal of mRFP repression. mRFP expression was calculated as specific fluorescence (relative fluorescence units/OD_600_) of the initially IPTG repressed ES114:JMP1189/psgRNA(RR1) strain normalized to the unrepressed ES114:JMP1189/psgRNA(RR1) control strain. Values reported reflect the mRFP repression at mid-log growth (OD_600_ = 0.4). Each point reflects the mean of 3 samples with their respective standard deviation from the mean. Statistics were done using a two-way ANOVA, with Šídák’s multiple comparisons test *: p < 0.05; ** p < 0.01; *** p < 0.001; **** p < 0.0001

To quantitatively test the gene repression capability of the PLlac0-1 driven dcas9 and sgRNA cassettes, we initially assayed the repression of mRFP fluorescence from the ES114:JMP1183 strain, which has both cassettes integrated at the attTn7 site along with a constitutive *mRFP* reporter gene. We concurrently assayed mRFP repression in the ES114:JMP1189 strain (which is isogenic to strain ES114:JMP1183 but without a sgRNA cassette) carrying a PLlac0-1 driven psgRNA (RR1) expression plasmid, and the psgRNA(NT) expression plasmid which has a 20-nt spacer designed to not match anywhere in the *V. fischeri* genome (Figs. 1 and 2). Both the genome integrated and plasmid borne sgRNA cassettes have the same 20-nt spacer (RR1) targeting the integrated constitutive mRFP reporter.

To assay the reversal of CRISPRi repression of the *mRFP* test gene when IPTG inducer is removed, cultures of ES114:JMP1189/psgRNA (RR1) grown overnight with 2 mM IPTG induction of repression were re-cultured without IPTG inducer and assayed for restoration of RFP expression (Fig. 2B).

### Functional testing of *luxC* repression

Bioluminescence is generated in *V. fischeri* when the autoinducer 3-oxo-C6 (Eberhard et al. 1981), activates the regulatory protein LuxR, which in turn activates transcription of the *luxICDABEG* operon. This operon contains *luxI*, (synthesis of the autoinducer 3-oxo-C6), *luxAB* (encoding dual-protein components luciferase enzyme), *luxCDE* (enzymes for synthesis of the luciferase aldehyde substrate) and *luxG*, a flavin reductase (Engebrecht et al. 1983; Engebrecht and Silverman 1984; Miyashiro and Ruby 2012; Nijvipakul et al. 2008). Since it has been shown that transcriptional repression caused by the steric hindrance of dcas9:sgRNA complex bound to a target gene is polar and blocks transcription of further downstream genes in an operon (Peters et al. 2016), we reasoned that targeting the *luxC* gene for repression would induce repression of the *CDABEG* genes of the operon and thus repression of bioluminescence.

**Fig. 3:**
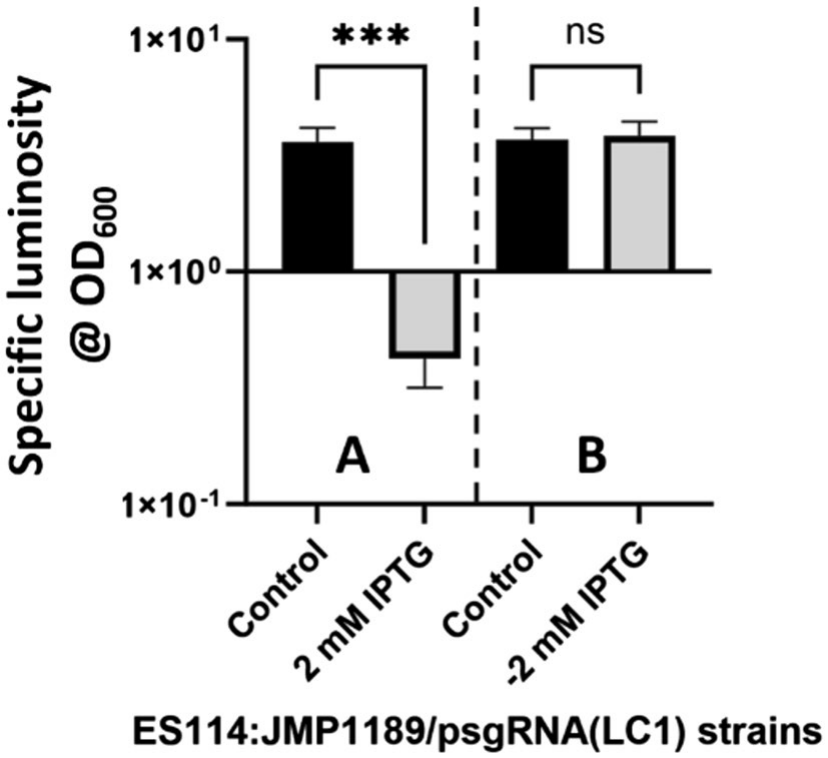
Repression and subsequent derepression of luminescence. **A** repression of specific luminosity (relative light units/OD_600_) in OD_600_ = 2.0 cultures of uninduced control strain ES114:JMP1189/psgRNA(LC1) and IPTG (2 mM) induced ES114:JMP1189/psgRNA(LC1). **B** derepression of specific luminosity (relative light units/OD_600_) in OD_600_ = 2.0 subcultures of the same strains grown in media with no IPTG. Shown are median specific luminosities of three independent experiments (biological replicates). Error bars indicate the standard deviation. T-tests showed significant differences in means for the IPTG repressed ES114:JMP1189/psgRNA(LC1) and control while there was no significant difference between the derepressed ES114:JMP1189/psgRNA(LC1) and control. (*** indicates P < 0.001; ns indicates P > 0.05)

The highest ranking prospective 20-nt targeting sequence abutting a 5’-NGG PAM sequence found near the initial sequence of the *luxC* gene (VF_A0923; Genbank: CP000021.2; 1440 bp) was identified by the CRISPy-web sgRNA design software (secondarymetabolites.org). Complementary *Bsa*I tailed oligos (LC1 F/R; IDT; Table 1) matching the chosen *luxC* (LC1) target site were annealed and cloned into the triple *BsaI* cloning site of plasmid psgRNA (*Bsa*I) via *BsaI* restriction/ligation generating plasmid psgRNA (LC1; Table 1). Plasmid psgRNA (LC1) was conjugated via tri-parental mating into *V. fischeri* strain ES114:pJMP1189. The time-course of inducible repression and subsequent de-repression of luminescence of cultured ES114:JMP1189 carrying plasmid psgRNA (LC1) is shown in Fig. 3.

**Table 1:** Maximum CRISPRi Repression Levels

Vector	Fold change under maximum repression
sgRNA(RR1)	~ 11
sgRNA(LC1) (in vitro)	~ 20
sgRNA(LC1) (in vivo)	~ 29
MMsgRNA(RR1)	~ 8
MMsgRNA(RR1,RR2)	~ 14
MMsgRNA(LC1)	~ 14
MMsgRNA(FA1)	~ 20

### CRISPRi repression of bioluminescence in the light organ

The utility of ES114JMP1189/psgRNA (LC1) for regulating bioluminescence production from *V. fischeri* in its host environment during symbiosis was demonstrated by inoculating newly hatched aposymbiotic *Euprymna scolopes* juveniles with ES114JMP1189/psgRNA (LC1) cells that were induced by IPTG before and during the initial colonization of the juvenile squid. By manipulating the time-course of *V. fischeri* luminescence production during initial colonization, we can probe the time-dependence of the “winnowing” selection that prevents non-luminous symbionts from maintaining a successful colonization (Visick et al. 2000). Earlier research using non-luminescent *luxA* knockout mutants established that while mutant *V. fischeri* could successfully initiate colonization of the light organ, by 48 h post colonization the number of symbionts remaining in the light organ declined precipitously. This decline in light organ symbiont numbers then continued till no mutants were left, leading to an unsuccessful symbiosis (Visick et al. 2000).

**Fig. 4:**
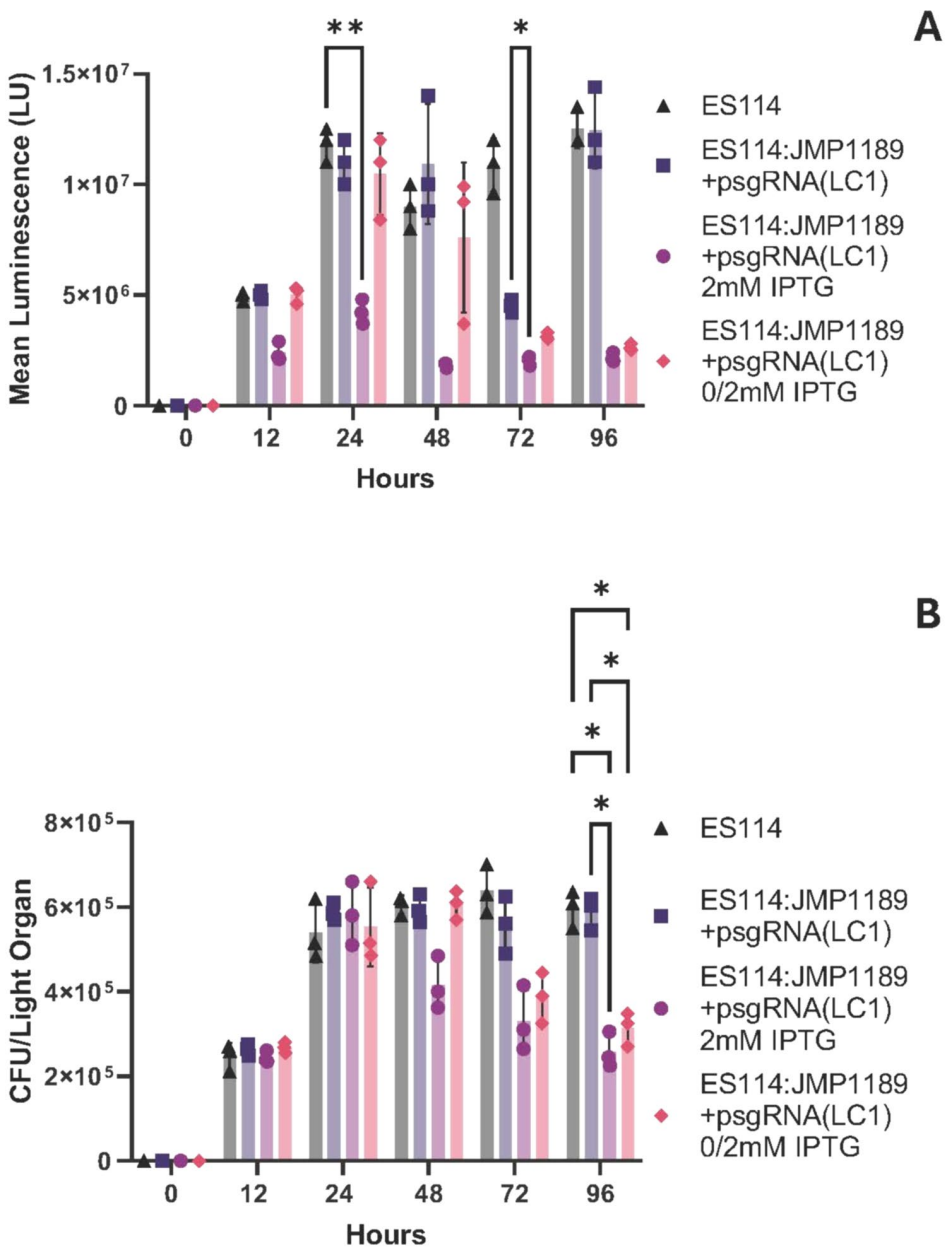
**A** Luminescence of juvenile squid infected with multiplex ES114 strains. Luminescence of juvenile squid infected with different ES114:JMP1189/psgRNA(LC1) cultures grown with and without IPTG induction. Hatchlings were placed in seawater containing either (•) WT ES114 control, (▪) ES114:JMP1189/psgRNA(LC1) with no inducer, (▴) ES114:JMP1189/psgRNA(LC1) induced with 2 mM before infection, (▾) ES114:JMP1189/psgRNA(LC1) with no inducer before infection, but with 3 mM IPTG added to seawater at 24 h post infection. Mean luminescence values are averages for 5 animals for each treatment. All values reported are mean values and error bars reflect the standard deviation from the mean. Statistics were done using a two-way ANOVA, with Šídák’s multiple comparisons test *: p < 0.05; ** p < 0.01; *** p < 0.001; **** p < 0.0001. **B** CFU in colonized juvenile squid. CFUs per light organ of juvenile squid infected with different ES114:JMP1189/psgRNA(LC1) cultures grown with and without IPTG induction. *E. scolopes* hatchlings were exposed to seawater containing either (•) WT ES114 control, (▪) ES114:JMP1189/psgRNA(LC1) with no inducer, (▴) ES114:JMP1189/psgRNA(LC1) induced with 2 mM before infection, (▾) ES114:JMP1189/psgRNA(LC1) with no inducer before infection, but with 3 mM IPTG added to seawater at 24-h post infection. CFU/LO counts were obtained by sacrificing juvenile squids at serial 24-h time points post infection and plating the homogenate on SWT agar plates. CFU/LO values are averages for 5 animals for each treatment. All values reported are mean values and error bars reflect the standard deviation from the mean. Statistics were done using a two-way ANOVA, with Šídák’s multiple comparisons test *: p < 0.05; ** p < 0.01; *** p < 0.001; **** p < 0.0001

In order to examine the ability to use IPTG-inducible CRISPRi gene repression of endogenous *V. fischeri* genes to modulate symbiont phenotypes during the course of symbiosis, we inoculated aposymbiotic hatchlings with ES114JMP1189/psgRNA (LC1) cells. Colonization was allowed to proceed for 24 h and subsequently IPTG was added to the hatchlings seawater to induce repression of luminescence. Luminescence levels from the hatchlings decreased by 48 h after infection and remained repressed out to 96 h, with a concomitant decline in light organ symbiont numbers (Figs. 4A, B), demonstrating the ability to successfully induce and maintain luminescence repression within colonized hosts.

### Multiplexing sgRNA plasmid vectors

**Fig. 5:**
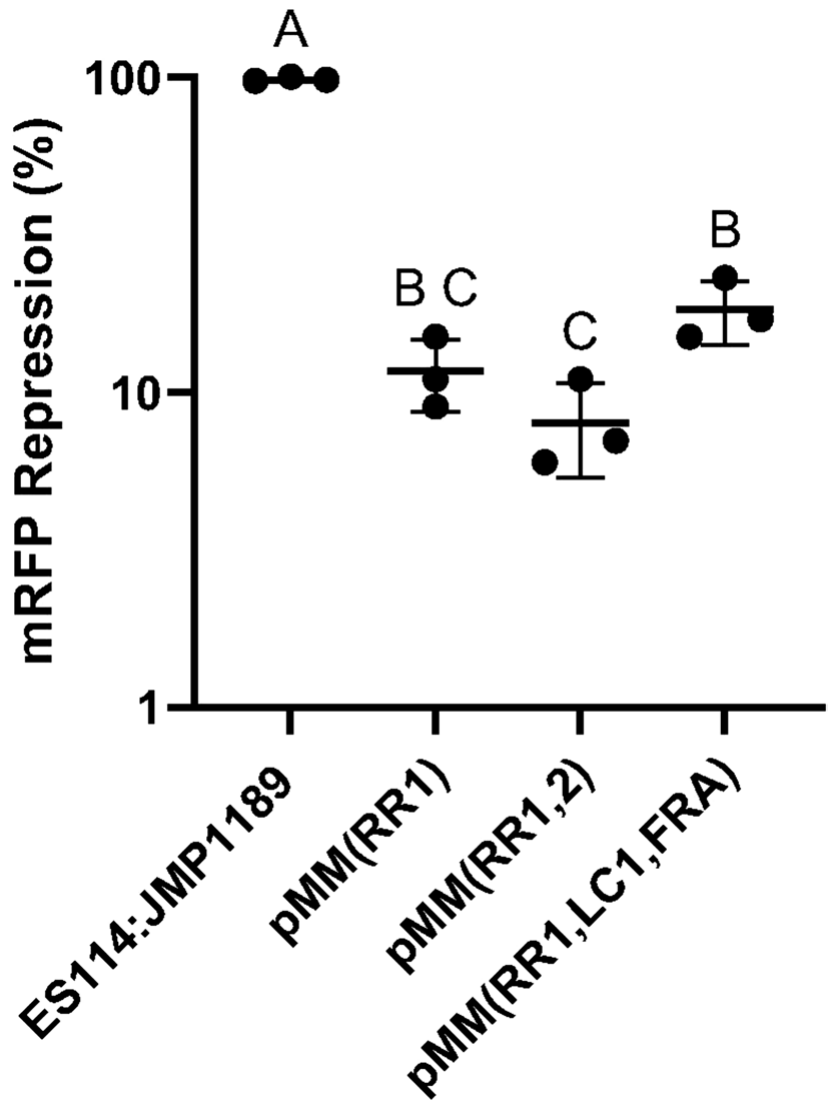
Multiplexed sgRNA repression of mRFP fluorescence. **A** Cultures grown with no IPTG; **B** Cultures grown with 2 mM IPTG induction of repression to an OD_600_ of 0.4, with triplicate 200 mL aliquots assayed for fluorescence in a 96-well multimodal plate-reader. Specific fluorescence (relative fluorescence units/OD_600_; excitation 584 nm, emission 607 nm) of the single and multiplexed sgRNA plasmid carrying strains was normalized to the control strain ES114:JMP1189 (mRFP + , no sgRNA). Columns indicate mean values of three independent experiments (biological replicates). Different letters on the abscissa denotate significant differences between groups according to the Tukey post-hoc comparison

As a final component of a generalized CRISPRi tool suite for *V. fischeri*, we investigated designs to combine the expression of multiple sgRNA transcripts from our psgRNA vector, which would allow for simultaneous repression of multiples genes-of-interest, or to target a single genetic locus with multiple sgRNA transcripts. Targeting multiple non-overlapping sgRNAs to a single gene can additively increase CRISPRi repression of transcription of that gene (Qi et al. 2013). We estimated that having two nearly homologous sgRNA cassettes in the same plasmid would lead to recombinational instability in *V. fischeri* (which has a generally high level of recombinational functionality (Thompson et al. 2007)) and therefore adopted a design that utilized nonhomologous sgRNA cassette component sequences that can be cloned into a single expression plasmid with minimal risk of recombination (Reis et al. 2019). A software sgRNA design tool (ELSA Calculator; De Novo DNA) was used to find combinations of non-homologous sgRNA cassette components (synthetic minimal promoters, RBS, sgRNA frameworks, transcription terminators) to enable the design of a sequence with three independent sgRNA cassettes that was cloned into our ES114:JMP1189 single sgRNA plasmid, replacing the sgRNA/*lacI* (*BsaI*) cassette. This generated the multimeric sgRNA cloning plasmid pMMsgRNA (3IIS). The three Type IIS spacer cloning sites can be sequentially restriction digested and cloned with tailed annealed oligomers, using the same protocol used for cloning spacer elements into the single psgRNA (*BsaI*) cloning plasmid. We generated multiple multiplexed sgRNA plasmids with combinations of 20-nt spacer targeting sequences and validated their function and utility in vitro. Two of the targeting spacers combined in multiplex format were the previously validated spacers for mRFP (RR1) and *luxC* (LC1), along with a spacer targeting the master regulator of the flagella production regulon, *flrA* (FA1) (Millikan and Ruby 2003; Wolfe et al. 2004). To test for enhancement of single gene repression through multiplexed 20-nt spacer targeting, we compared the repression of the test mRFP reporter in ES114:JMP1189 using the single 20-nt pMMsgRNA (RR1) with a version expressing two sgRNA to mRFP (pMMsgRNA (RR1, RR2). A triple sgRNA multiplex with targeting spacers for *mRFP*, *luxC*, and *flrA* was also able to cause significant fluorescence repression (Fig. 5).

**Fig. 6:**
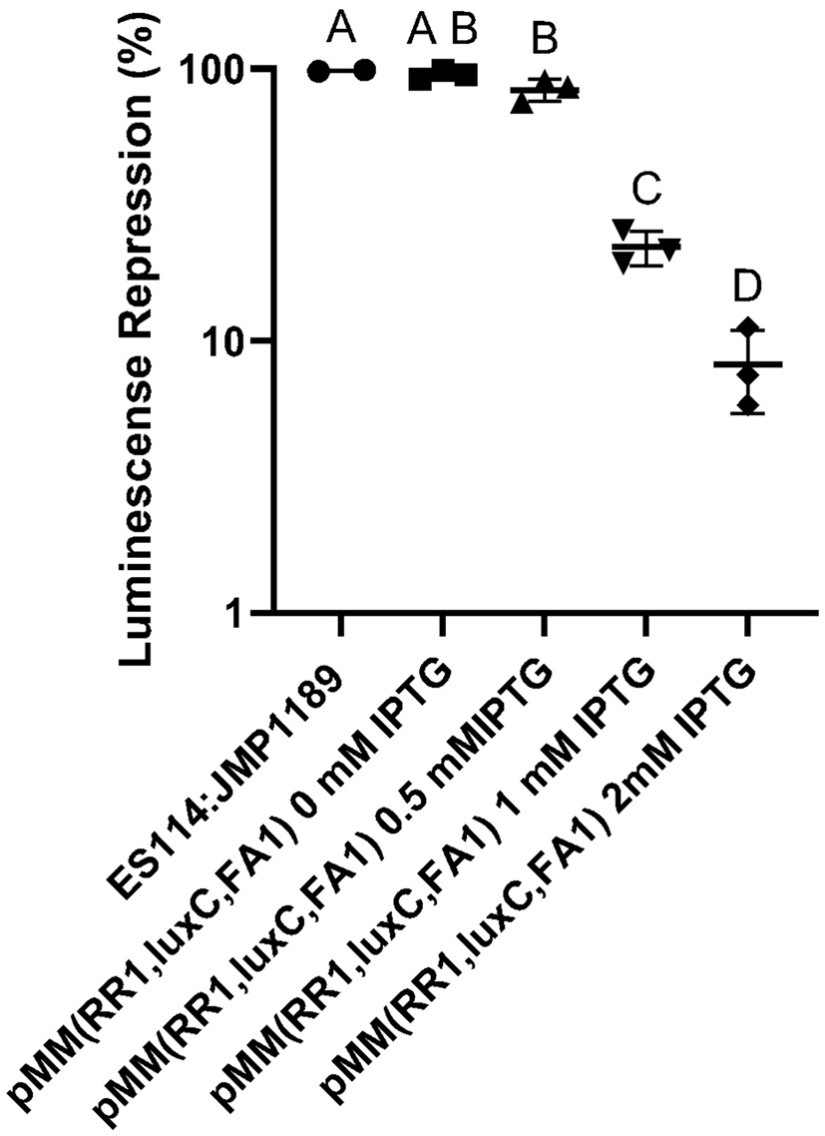
Multiplexed sgRNA repression of luminescence. ES114:JMP1189/pMM(RR1, LC1, FA1) cells were sub-cultured in SWT with the indicated levels of IPTG inducer and grown to an OD600 of 2.0, with triplicate 200 mL aliquots assayed for luminescence in a 96-well multimodal plate-reader. The specific luminescence (relative luminescence units/OD_600_) of the single and multiplexed sgRNA plasmid carrying strains was normalized to the control strain ES114. Columns reflect mean values; error bars indicate standard deviations. Different letters on the abscissa denotate significant differences between groups according to the Tukey post-hoc comparison

**Fig. 7:**
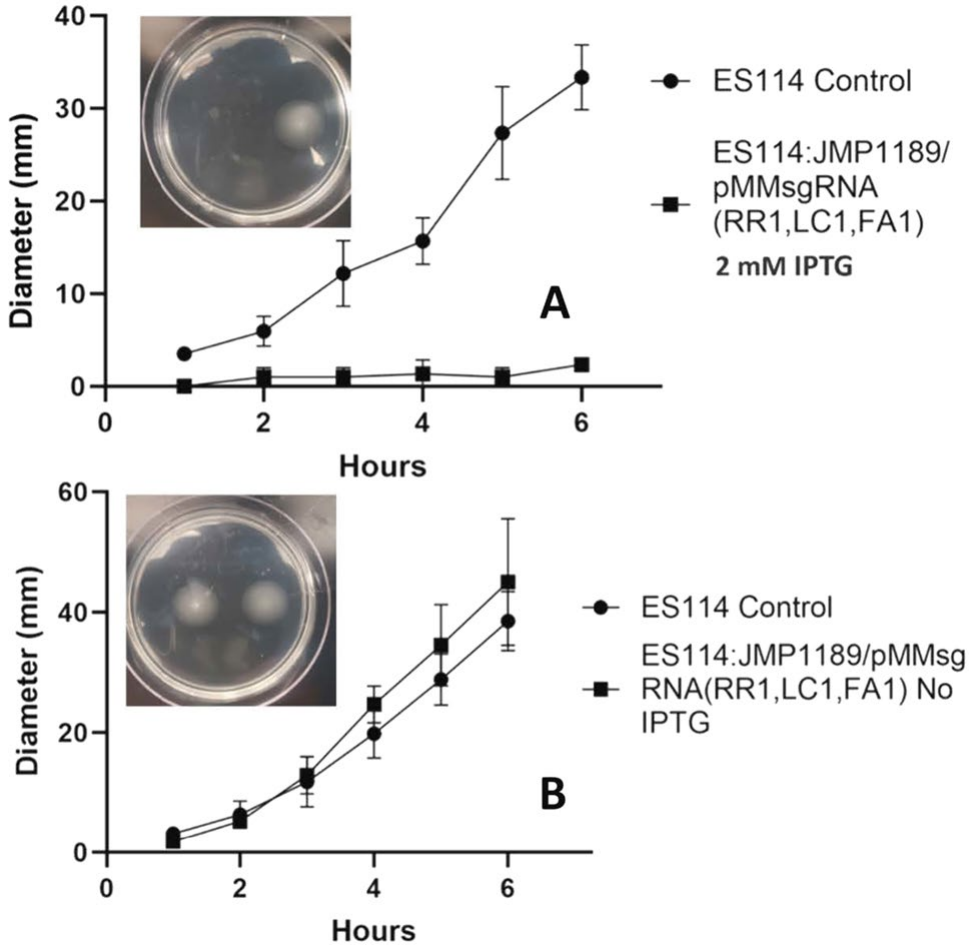
Motility of ES114:JJMP1189/pMMsgRNA(RR1,LC1,FA1). **A** Migration of ES114:JJMP1189/pMMsgRNA(RR1,LC1,FA1) sub-cultured with 2 mM IPTG and spotted on TBS-Mg^2+^ agar with 2 mM IPTG. **B** Migration of ES114:JJMP1189/pMMsgRNA(RR1,LC1,FA1) sub-cultured without IPTG and spotted on TBS-Mg^2+^ agar. Inserts show representative motility plates measured at 6 h. Statistics were done using a two-way ANOVA, with Šídák’s multiple comparisons test *: p < 0.05; ** p < 0.01; *** p < 0.001; **** p < 0.0001.Error bars reflect standard deviations. *: p < 0.05; ** p < 0.01; *** p < 0.001; **** p < 0.0001

This triple multiplex sgRNA plasmid was also assayed for its ability to repress luminescence and bacterial motility. Figure 6 shows titratable repression of luminescence in ES114:JMP1189/pMMsgRNA (RR1, LC1, FA1) cells in culture supplemented with 0–2 mM IPTG. Functional testing of CRISPRi repression of the third targeted gene in the multiplex sgRNA plasmid, *flrA*, revealed severe repression of cell motility in a standard soft-agar motility assay (Fig. 7).

## Discussion

dCas9-based CRISPRi inhibits transcription in bacterial systems during mRNA transcription initiation or elongation through steric hindrance, physically blocking RNA polymerase progression (Banta et al. 2020a, b). The targeting sequence requirements for the popular *S. pyogenes* dcas9 (Spdcas9)—*i.e.*, a 20-nt target sequence next to an upstream 5’-NGG PAM sequence (Jinek et al. 2012) suggests that the vast majority of bacterial genes (or other loci) will be accessible to dcas9/sgRNA binding. The *V. fischeri* CRISPRi system of vectors developed in this work is a relatively streamlined general purpose tool for programmable control of either single or multiple gene(s) expression which is highly flexible and modifiable for diverse applications. The system can efficiently repress both exogenous (*mRFP*) and endogenous (*luxC*, *flrA*) genes in *V. fischeri*, both when grown in culture and within their symbiotic host.

Our *V. fischeri* CRISPRi system uses stable plasmid vectors (no antibiotic selection pressure required to maintain the plasmid; Dunn et al. 2006) for expression of programable sgRNA transcripts in conjunction with an *attTn7* site integrated *dcas9* expression cassette. The Tn7 transposases recognize a site in the highly conserved *glmS* gene (essential in *E. coli* (Milewski 2002) and likely essential in nearly all bacteria), and inserts immediately downstream of *glmS* into the *attTn7* site, such that no gene is disrupted with no discernable fitness cost to the host (Peters and Craig 2001). Transposition occurs exclusively into *attTn7*, and not into other locations (Peters and Craig 2001), and allows for the stable integration of genes in single copy. Transgene expression from the *attTn7* site has not been seen to have, nor been affected by, any polar effects with neighboring loci (Chang et al. 2024).

Design and construction of sgRNA plasmids targeting any gene/locus-of-interest is a simple process of Type IIS restriction cloning of 20-nt spacer sequences (as annealed pairs of oligonucleotides) into the psgRNA (*BsaI*) cloning plasmid that are complementary to the non-template strand of the target gene/locus and adjacent to the required PAM (5’-NGG; Fig. 1). Software packages have been developed and are available on-line for identifying such target sequences in sequenced genomes (Blin et al. 2016; www.synthego.com; https://crispy.secondarymetabolites.org). Target sequences can also be found by manually searching of the gene(s)-of-interest sequence, but the sgRNA software performs additional laborious quality checks such as searching the *V. fischeri* genome for off-target sequences that have unintended full or partial matches to any putative 20-nt targeting sequences (Blin et al. 2016, 2020).

Initial function validation of our CRISPRi system used a sgRNA plasmid targeting a chromosomally integrated *mRFP* gene for repression. IPTG inducer supplementation of the media of exponentially growing mRFP tagged ES114 cells (ES114:JMP1189) carrying the psgRNA (RR1) plasmid showed titratable repression of mRFP reporter fluorescence at IPTG concentrations in a range of 0.05 to 2.0 mM/mL (Fig. 2). We observed significantly greater repression from this plasmid-based system, compared to the fully integrated CRISPRi strain ES114:JMP1183. This is consistent with reports that sgRNA transcript levels can be limiting in CRISPRi applications (Fontana et al. 2018), (Peters et al. 2019; Byun et al. 2023); our multicopy plasmid (~ 10 copies/cell) sgRNA expression provides a higher molar ratio of sgRNA transcripts to dcas9 enzymes in the cells, which would facilitate higher rates of binding and inducing steric repression (Banta et al. 2020a, b). To ensure that there was a saturating amount of *lacI* repressor protein present in the cells to bind to the multiple PLlac0-1 promoters on the psgRNA plasmids, a *lacI* expression cassette was included on each sgRNA expression plasmid, in addition to the *lacI* expression cassette included in the *attTn7* integrated *dcas9* cassette.

### CRISPRi modulation of symbiont gene expression and phenotype during host colonization

Work in genetic tool development for marine bacteria is continually expanding, reflecting their wide-spread associations in aquatic microbiomes. α-proteobacteria and γ-proteobacteria symbionts have been subject to genetic modification for research in phytoplankton (Sunagawa et al. 2015), coral (Bourne et al. 2016), tubeworm (Vijayan et al. 2019), and sepiolid squid (Dunn et al. 2006; Visick et al. 2018) symbiotic systems. Recently, a novel CRISPRi plasmid tool was used to demonstrate the ability to modulate gene expression in the symbiont marine bacterium *Pseudoalteromonas luteoviolacea* during the course of its symbiosis with the model tubeworm, *Hydroides elegans* (Alker et al. 2023). In their study, CRISPRi repression of the *macB* gene, which is essential for inducing host tubeworm metamorphosis, was shown to lead to significantly reduced levels of metamorphosis in the host.

To demonstrate the utility of our CRISPRi system for modulating endogenous *V. fischeri* gene expression in symbionts within the host squid, we chose to target bioluminescence production during squid hatchling colonization. The production of bioluminescence by *V. fischeri* during the initial colonization of host tissues, and latter during the maintenance of the symbiosis within the mature light organ, is intricately coordinated by multiple environmental conditions and cues within host microenvironments (Pan et al. 2015; Chavez-Dozal et al. 2012; Nourabadi and Nishiguchi 2021), to ensure that bioluminescence is rhythmically produced in a diurnal pattern (Schwartzman et al. 2015). Bioluminescence produced by *V. fischeri* has also been found to act as a cue for changes in the tissue development of the immature colonized light organ (Visick et al. 2000), with hosts colonized by non-luminescing mutant *V. fischeri* strains failing to undergo normal light organ epithelial tissue maturation. These “dark” mutants are also actively rejected by the host after initial colonization, implying that the regulation of bioluminescence by microenvironmental conditions/cues (pH, oxygen saturation, nutrient status, cell density/quorum signaling) found within the host is also accompanied by an ability of the host to monitor the proper bioluminescence response from the symbionts (Visick et al. 2000; Chavez-Dozal et al. 2012; Nourabadi and Nishiguchi 2021; Pipes and Nishiguchi 2022). Inducible CRISPRi repression of bioluminescence provides an experimental tool for over-riding the normal regulation of bioluminescence during colonization; in our proof-of-concept application we were able to repress luminescence 48 h after initial colonization of hatchlings and recapitulate the decline in symbionts seen during initial colonization with mutant dark symbionts (Visick et al. 2000). Currently we are unable to extend the time course of our experiments past 96 h as we have been unsuccessful in raising the juvenile squid past this point. If this husbandry hurdle can be crossed, future studies that follow our CRISPRi repressible luminescence strains past our current 96 h time-course will be able to reveal the effects of reversing the repression and restoring luminescence in the remaining symbionts.

Bioluminescence in *V. fischeri* is produced by the luciferase enzyme, a heterodimer of LuxA and LuxB proteins. *luxA* and *luxB* are members of the *lux* operon (*luxCDABEG*). The LuxR-AHL transcriptional activator complex binds to the promoter region of the operon and activates polycistronic transcription of the genes.

CRISPRi repression of any of the genes in the *lux* operon would lead to loss of bioluminescence resembling the mutations (natural and artificial) in different *lux* operon genes that result in “dark” mutant strains (O’Grady and Wimpee 2008). We chose to target the *luxC* locus because it is the first gene in the operon and steric hindrance at the *luxC* locus would produce a polar repression (Peters et al. 2016) of the remaining genes in the operon (*luxDABEG*). Functional validation of in vitro bioluminescence repression was conducted using strain ES114:JMP1189 carrying the psgRNA (LC1) plasmid. The maximum level of repression was different than that found in validation tests of the mRFP targeting plasmid psgRNA (RR1)—this is consistent with work showing the level of repression achieved using CRISPRi being gene-specific (Banta et al. 2020a, b), and the time course required for loss of function through transcriptional repression reflecting the stability (half-life) of the protein(s) being repressed, as well as the growth rate of the cells (lowering functional levels of the repressed protein through dilution during cell division) (Qi et al. 2013). The times observed for maximally induced repression, and the lifting of that repression to initial levels in the mRFP test strain could serve to guide gene function experiments completed in similar cell culture conditions but are not as useful to guide research using timed CRISPRi repression of genes during *V. fischeri* host colonization. The growth pattern and phenotype of cells in an established light organ colonization are notably changed compared to the growth pattern, and phenotype, of the same cells grown in culture (Chavez-Dozal et al. 2014; O’Shea et al. 2005). During the rhythmic diel cycle of the light organ, 95% of the symbionts in the light organ are expelled at dawn. The remaining cells repopulate the light organ crypt microenvironments through rapid growth, reaching maximal cell density levels by noon (Norsworthy and Visick 2013). The rate of growth then diminishes abruptly (generation time increasing from ~ 0.5 h to 10–18 h) and cells can be observed to become reduced in size and non-flagellated (Ruby and Asato 1993). Since accurate estimations of the rate of timing of repression and de-repression of the *luxC* gene in the light organ microenvironment could not be generated in culture, the timing of induction of CRISPRi repression in the colonized squid experiments had to be determined empirically.

ES114:JMP1189 carrying psgRNA (LC1) was used to inoculate abiotic hatchling squid to demonstrate the ability of inducible timed repression of bioluminescence in vivo to probe the temporal monitoring of symbiont bioluminescence by the host (Fig. 5). The addition of IPTG to the hatchlings seawater at 24 h post inoculation was able to induce and maintain repression of bioluminescence from the symbionts to levels that triggered the same pattern of loss of symbiont numbers seen during early stages of colonization with dark *lux* mutants (Koch et al. 2014). Notably, the drop in CFU/LO at 55 h post inoculation followed the same pattern as seen at 24 h in “dark” ES114:JMP1189 carrying psgRNA (LC1) that were fully IPTG induced at inoculation. Our results demonstrate the utility of inducible CRISPRi repression in probing the temporal distribution of regulatory conditions (bioluminescence) and responses (symbiont rejection) during the symbiosis.

### Multiplex plasmid design

CRISPRi systems that target single genes enable relatively rapid and facile transcriptional repression for forward genetics studies, avoiding laborious mutant strain construction methodologies that result in altering the native regulation of the gene (Qi et al. 2013; Peters et al. 2019; Mimee et al. 2015; Rock et al. 2017; Jiang et al. 2024). Simultaneous CRISPRi targeting of multiple genes in the same cell (multiplex CRISPRi) (Larson et al. 2013; Qi et al. 2013; Peters et al. 2016; Zhao et al. 2016) avoids the even more laborious construction of multiple-deletion strains. Multiplex CRISPRi systems have been demonstrated in various bacterial species using a variety of methods to express multiple sgRNAs—including crRNA arrays (Kim et al. 2017), multiple arrayed independent sgRNA cassettes (Qi et al. 2013; Peters et al. 2016), and nonrepetitive extra-long sgRNA arrays (ELSAs; Reis et al. 2019).

Our novel *V. fischeri* multiplex sgRNA plasmid uses an ELSA cassette for the simultaneous expression of three sgRNAs. Each sgRNA cassette within the multiplex plasmid had a unique strong synthetic constitutive promoter, a 20-nt spacer sequence targeting an independent genetic locus, functional sequence variant sgRNA framework sequences, and unique transcription terminators and spacing sequences. With this design, no sequence part in the three sgRNA cassettes shares more than 17 bp homology with another part. This lack of homology, compared to the > 90% sequence homology between repeated sgRNA cassettes has two advantages: (1) plasmids with repeated sequences (areas of homologous sequence) can recombine in highly recombinogenic bacteria like *V. fischeri* (Lin et al. 2018), altering the content of the plasmid or rendering it unstable for replication, and (2) Synthetic dsDNA construct services cannot synthesize sequences containing areas with homologous repeats (Reis et al. 2019). Notably, the three unique sgRNA cassettes could not use the same PLlac0-1 inducible promoter used in the single sgRNA cassette plasmids (excessive sequence homology) and having three different inducible promoters would make it logistically difficult to utilize. Inducibility of the multiplexed CRISPRi system comes from the single PLlac0-1 promoter driving the integrated dcas9 cassette in the parent ESS114:JMP1189 strain.

We observed higher mRFP repression from the multiplex plasmid with two non-overlapping mRFP spacer sequences compared to the multiplex plasmid with a single mRFP spacer sequence, demonstrating the ability of a multiplexed sgRNA plasmid to generate increased target gene repression by providing two simultaneous sites of steric hindrance (Qi et al. 2013). The ability of a multiplex sgRNA plasmid to simultaneously repress three separate genes was demonstrated using assays for mRFP fluorescence, bioluminescence, and motility on soft-agar plates. The *flrA* regulatory gene was chosen as a gene-of-interest in this study because it has also been shown in prior research (Chavez-Dozal et al. 2012; Millikan and Ruby 2003; Shrestha et al. 2022) to be the master transcription activator of the flagella production regulon (which contains other non-flagella associated operons; Millikan and Ruby 2003). In *V. fischeri*, flagellar gene regulation is controlled in a multilevel cascade (Norsworthy and Visick 2013), with FlrA activating early flagellar genes, including *flrBC* and *fliA.* Expression of the late genes, multiple flagellar filament and motor proteins, is regulated in a sequential cascade first by FlrBC and then FliA (Aschtgen et al. 2019). *flr*A is necessary for initial colonization of the light organ, but its expression is repressed via unknown signals and cues in the light organ where most symbionts appear non-flagellated (Ruby and Asato 1993), only to be re-expressed shortly before symbionts are expelled at dawn (Norsworthy and Visick 2013). When insertional (DM126, DM127, and DM128) and partial deletion (DM159) mutations of the *flrA* gene were constructed they were shown to be non-motile in soft agar motility plates (Millikan and Ruby 2003). Additional Δ*flrA* knockout mutants in *V. fischeri,* generated by targeted homologous recombination, were also found to be non-motile in soft-agar (Visick et al. 2018).

The work presented demonstrates proof-of-principle utility of a CRISPR interference suite of vectors and strains for inducible repression of expression of both exogenous and endogenous genes-of interest via simple targeting with target sequence complementary 20-nt spacer sequences cloned into sgRNA expression plasmid vectors. The single IPTG inducible sgRNA expression plasmids, used with a common genomically integrated IPTG inducible dcas9 ES114 strain, can repress *V. fischeri* gene expression in a titratable and reversable course in culture, as well as in symbionts colonizing juvenile host squid. A multiplex version of the sgRNA plasmid, though not inducible itself, provides the capability for elevated repression of a single gene via multiple independent complementary targeting spacer sequences to that gene, or can simultaneously target up to three genes for repression. CRISPR interference is a powerful, flexible general genetic programming tool for research in the *V. fischeri*—*E. scolopes* symbiosis model system, complementing and extending the current toolbox of gene modification methodologies and allowing for determining the exact timing of the genetic cross talk between hosts and their beneficial microbes.

## Materials and methods

### Strains and media

Strains and plasmids used in this study are listed in Table S1. Oligos, and synthetic dsDNA constructs used in this study are listed in Table S2. *Escherichia coli* strains DH5α and pir + competent GT115 (InvivoGen) were used for plasmid maintenance, cloning, and conjugation. *V. fischeri* strain ES114 was grown aerobically using various media at 28 °C. For molecular biology applications and fluorescence assays, *V. fischeri* cells were cultured in Luria–Bertani high salt (LBS) containing 10 g of tryptone, 5 g of yeast extract, 20 g of NaCl, and 20 mM Tris-hydrochloride (Tris–HCl, pH 7.5) per L of sterile water. All squid colonization and bioluminescence assays used seawater tryptone (SWT), which contained 5 g of tryptone, 3 g of yeast extract, and 700 mL of artificial seawater (ASW) per L of sterile water. *E. coli*, aerobically grown at 37 °C, was cultured in Luria–Bertani (LB) media, which contained 10 g tryptone, 5 g yeast extract, and 10 g NaCl. Agar was added to the media at 15 mg/mL for plating. Where appropriate, the antibiotics chloramphenicol and kanamycin were added to growth media at 1–3 and 100 μg/mL, respectively, for *V. fischeri* with ampicillin, chloramphenicol and kanamycin added to growth media at 100, 30, and 100 μg/mL respectively, for *E. coli*. Thymidine (0.3 mM) was used to supplement LB media for culturing of the thy-auxotroph DH5α strain carrying conjugation helper plasmid pEVS104. IPTG (Isopropyl ß-D-1-thiogalactopyranoside) (GoldBio), for CRISPRi induction was added to media when appropriate as indicated.

Artificial seawater for use with *E. scolopes* hatchlings was made using ddi H_2_0 (1L) with 30 g Instant Ocean® marine salt (Instant Ocean Spectrum Brands) and 10 g Marine Mix® marine salt (Bulk Reef Supply). Artificial saltwater was aerated with aquarium air-pumps and air-stones.

### Molecular cloning

All molecular cloning reagents (restriction enzymes) are from NEB. Plasmid extraction Plasmid Miniprep Kits (ZymoPURE™) from Zymogen. Primers were ordered from IDT DNA. Synthetic dsDNA constructs were ordered from Twist Biosciences.

### Design of a *V. fischeri* CRISPRi suite of vectors

Our experimental goals were to create an inducible CRISPRi system able to target and repress single or multiple genetic loci in *V. fischeri*, both in culture and in symbiosis with their squid host. These goals lead to several design constraints-

- The dcas9 and sgRNA components must be stably retained through prolonged growth periods—up to several days of growth in infected squid. The use of antibiotic selection to enforce retention of plasmid vectors is problematic because of potential toxicity of common antibiotic selective agents with the squid host, and incomplete penetration of antibiotics within the light organ crypts (Dunn et al. 2006).
- The inducible promoter(s) used to control expression of CRISPRi components should have minimal leaky expression when uninduced and the inducer used with the system, when added to sea-water, must be non-toxic to the squid host, and be able to penetrate the crypts of the light organ.
- Express sgRNA in excess of dcas9 to ensure that the proportionately lower concentration of individual sgRNA transcripts produced from a multiplex (three sgRNA cassette) vector can saturate the expressed dcas9.

Successful CRISPRi systems have been developed that genomically integrate both *dcas9* and sgRNA (Liu et al. 2017; Peters et al. 2016), as well as systems that contain *dcas9* and sgRNA on dual or single stable plasmid vectors (Rachwalski et al. 2024; Tan et al. 2018). For our goals though, heterologous systems, with an integrated *dcas9* and plasmid expressed sgRNA (Rousset et al. 2018) fit all of the design constraints.

- *dcas9* cassettes have been previously integrated into the *V. fischeri* genome, at the intergenic *attTn7* Tn7 transposon site, via conjugation of an integrating plasmid vector(Geyman et al. 2024). Tn7 integrated *dcas9* cassettes have been shown to be stably retained through > 50 generations of antibiotic selection-free growth in culture while maintaining *dcas9* expression (Peters et al. 2019). Stable *V. fischeri* plasmids have been developed, derived using the native pES213 plasmid origin of replication, which are stable (Dunn et al. 2005) during colonization of the squid. pVSV105 is a widely utilized conjugatable pES213 derived plasmid (copy number of 9.4 ± 2.4) which has been found to be ≥ 99% retained in *V. fischeri* after nonselective growth in culture for 60 generations, or 72 h of squid colonization (Dunn et al. 2006).
- IPTG inducible promoters, such as the Plac, Pma, and PLlac0-1 promoters, have been successfully used in *V. fischeri,* both in culture and in squid hosts (Geyman et al. 2024; Visick et al. 2018). IPTG is non-toxic to the squid host and penetrates the light organ crypts (Visick et al. 2018). Dual PLlac0-1 promoters controlling both *dcas9* and sgRNA expression were recently used in the *attTn7* integrating Mobile-CRISPRi system to repress the *rpoB* essential gene in *V. fischeri.* The system exhibited negligible non-induced *dcas9* expression, while showing significant induced *rpoB* repression (Geyman et al. 2024).
- The multicopy stable pVSV105 plasmid provides ~ 10 sgRNA expression cassettes for every single genomic dcas9 expression cassette. Using the same PLlac0-1 inducible promoter for both provides coordinated induction of both components, with an excess of sgRNA. This excess of sgRNA transcripts, due to using the same promoters for *dcas9* and sgRNA while having the *dcas9* integrated and the sgRNA expressed from multiple plasmids, is an elegant way to ensure that in the multiplex version of the sgRNA plasmid there is enough of each individual sgRNA transcript to saturate the expressed dcas9 (Luo et al. 2015; Reis et al. 2019; Vigouroux and Bikard 2020). In a CRISPRi study in *Zymomonas mobilis* using independently inducible promoters for dcas9 and sgRNA expression, it was noted that the concentration of sgRNA relative to the concentration of dcas9 was a limiting factor in CRISPRi repression (Banta et al. 2020a, b).

### Tn7 transposon integrated *dcas9* expression *V. fischeri* strain construction.

A Mobile-CRISPRi Tn7 transformation donor plasmid developed for conjugational transfer into γ-proteobacteria (pJMP1183-Addgene, #119254; (Peters et al. 2019)) was used to transfer a mRFP (monomeric red fluorescent protein) expression cassette (for use as a repression target in testing), the *lacI* gene needed for IPTG induction, an IPTG inducible (PLlacO-1 promoter) *dcas9* cassette, an IPTG inducible (PLlacO-1 promoter) sgRNA (RR1) expression cassette targeting the introduced mRFP gene for repression, and a KanR selection marker into *V. fischeri* ES114 at the *attTn7* locus to generate strain ES114:pJMP1183. We also separately transferred pJMP1189 (Addgene, #119257; Peters et al. 2019) to introduce an IPTG inducible (PLlacO-1 promoter) dcas9 cassette, the *lacI* gene needed for IPTG induction, a KanR selection marker, and an mRFP expression cassette (for use as a repression target in testing, and later as a fluorescent cell tag) into *V. fischeri* ES114 at the *attTn7* locus to generate strain ES114:pJMP1189. These were transferred to *V. fischeri* ES114 by tetra-parental conjugation with the helper *E. coli* strains CC118 λpir + /pEVS104 thy- (pESV104 contains conjugative transfer genes and is a thymidine auxotroph) and BW25141/pJMP1039 (expresses Tn7 transposase; see Table S1). (Christensen et al. 2020). Briefly, *E. coli* donor strains (carrying Tn7 transposon plasmids pJMP1183, and pJMP1189, respectively) and *E. coli* helper strain Dh5a thy- carrying pEVS104 (Eric Stabb and Ruby 2002) along with a second *E. coli* helper strain carrying transposase (pJMP1039, Addgene # 119239; Peters et al. 2019) were cultured individually overnight in 5 mL LB media at 37 °C with appropriate antibiotics and additives*. V. fischeri* strain ES114 was cultured overnight (14 h) in SWT media at 28 °C with shaking. All cultures were then sub-cultured (100 μL aliquots into 5 mL appropriate media) and grown at their respective temperatures with shaking for 3–4 h. 250 μL of each of the four cultures were combined into a 1.5 mL microcentrifuge tube (Eppendorf). A 250 μL donor only control from each of the three *E. coli* cultures was placed in 1.5 mL microcentrifuge tubes, and a 250 μL recipient-only (ES114) control was placed in a 1.5 mL microcentrifuge tube. Cells were then pelleted at 8000 rpm for 5 min in a microcentrifuge (Eppendorf), and the supernatant discarded leaving 10–20 μL liquid in the tube. The pellets were resuspended in this liquid, and 100 μL of each tube was spotted in the middle of a pre-warmed LBS agar plate. After the liquid had absorbed into the plates, they were inverted and incubated overnight in a 28 °C incubator. In the morning, the colony was scraped off with a sterile pipette tip and the cells resuspended in 1 mL LBS by vortexing briefly. 100 μL aliquots of each cell culture were spread onto antibiotic containing LBS agar plates for selection of *attTn7*-site integrated transformed colonies (in the case of pJMP1183 and pJMP1189 this is Kanamycin, 100 μg/μL). Plates were incubated at 18 °C to repress growth of donor *E. coli,* and colonies of transformed *V. fischeri* selected when they appeared after 1–3 days. 20 of these colonies were isolated onto master LBS Kan 100 μg/mL agar plates and tested for the presence of integrated CRISPRi transposon construct by patching the master plate colonies on LBS with ampicillin agar and LBS kanamycin agar and incubating the plates at 28 °C overnight. Strains that have integrated the transposon segment and no longer retain the donor plasmid are AmpR-, KanR+. Further confirmation of genomic integration at the *attTn7* site is completed colony PCR using primers flanking the *attTn7* insertion site.

### sgRNA expression plasmids construction

#### Single sgRNA cassette expression plasmids

Single sgRNA expression plasmids were made using the *V. fischeri* stable shuttle vector pVSV105 as a template for PCR amplification of a vector backbone segment containing the Vf *oriV*, an i*ncP oriT*, the R6Kg *oriV*, and a CamR cassette, using tailed primers to provide ~ 30 bp homologous end overlap to the insert to be cloned. For the single (no targeting spacer sequence) sgRNA plasmid psgRNA, the empty sgRNA (triple-*BsaI*) expression cassette from plasmid pJMP1339 (Addgene #119271) was PCR amplified with tail primers that provided ~ 30 bp homologous end overlap to the pVSV105 backbone PCR amplicon (See Table S2 for sequences). These pieces were cloned into *pir* + GT115 competent *E. coli* using the NEBuilder® HiFi DNA Assembly Cloning Kit (NEB; Cat. # E5520S), following NEB protocol. Clones were selected as colonies on chloramphenicol antibiotic plates and the presence of correct constructs was confirmed by colony PCR with primers spanning cloning joints (Table S2).

### Constructing 20-nt targeting sequences

The highest ranking candidate sgRNA spacer 20-nt sequences targeting the *mRFP1, mRFP2*, *luxC*, and *flrA* genes were found with the CRISPy-web on-line sgRNA software (CRISPy-web; secondarymetabolites.org), using the *V. fischeri* ES114 reference genome (GenBank: GCA_000011805.1) to identify optimal dcas9 targeting sites that have no potential off-target sites (sequences with more than 9/20 homologous bases to the spacer sequence). Candidate spacers closest to the 5’ end of the ORFs were always chosen since studies of the efficacy of sgRNA targeting locations along several gene sequences suggest that achieving high efficacy gene knockdown in *Vibrio* species necessitates targeting toward the 5′ end of the open reading frame (Geyman et al. 2024). Additional sequence was added to the 5’ end of these sequences to provide compatible single strand overhangs that match the restriction sites of the sgRNA plasmid spacer cloning site (*Bsa*I for the single sgRNA plasmids, and *Bbs*I, *Bsa*I, and *Bae*I for the multiplex plasmid spacer cloning sites. Complementary pairs of annealed 5’-phosphorylated oligos with these 20-nt spacer + single strand overhangs were ordered from IDT DNA. See Table S2 for sequences of oligos.

### Cloning 20-nt targeting sequences into empty sgRNA (triple-*BsaI*) plasmids

The annealed oligo spacer sequences targeting the *mRFP*, *luxC*, and *flrA* genes were cloned into the triple-*Bsa*I targeting sites of psgRNA plasmids via ligation of annealed oligos (Brooks et al. 2014). 2 μL of a 1:20 dilution of the annealed oligos was ligated to 100 ng BsaI*-*digested psgRNA for 30 min at room temperature following the manufacturers protocol (NEB.T4 Ligase, M0202). The ligation mixture was used to transform ChemiComp GT115 Competent *E. coli* (Invivogen, Cat. Code gt115-11, 0.1 mL aliquots) following the manufacturers protocol. Briefly, 100 μL of frozen GT115 cells were thawed on ice and then transferred into a chilled 1.5 mL tube. 5 μl of the ligation reaction was added to the cells, mixed, and returned to ice for 30 min. The cells were heat shocked in a 42 °C water bath for 30 s and then returned to ice for two minutes. 900 μL of room temperature SOC medium was added to the reaction and the cells were incubated for one hour at 37 °C with shaking (250 rpm). 200 μL of the reaction was spread on LB agar plates with 50 μg/mL chloramphenicol and incubated overnight at 37 °C. 20 isolated colonies were collected the next day and spotted with a sterile pipette tip on a master plate (LB agar plates with 50 ug/mL chloramphenicol) which was grown overnight at 37 °C. When spotting the master plate, the pipette tip was also used to transfer some of the colony to a 50 μL PCR reaction mix in a 0.2 mL PCR tube. Colony PCR was performed using Q5 Hi-Fi 2X MasterMix (NEB, Cat. # M0492S) per the manufacturers protocol. The thermocycling conditions for the reaction were: Initial denaturization 98 °C 30 s, 25 cycles @ 98*C denaturation 10 s, annealing 66–70 °C (primer dependent, see Table S2 for primer sequences) 30 s, extension 72*C 30 s. A final extension at 72*C 2 min, and then held at 4 °C. 10 μL of each colony PCR reaction was mixed with 2 μL 6X Purple Loading Dye (NEB; Cat. #B7024S) and run on a 1% agarose gel in TAE buffer at 75 V until the loading dye front was ~ ¾ down the gel. 1 μL of 1 kb Plus DNA Ladder (NEB; Cat. #N3200S) mixed with 1 μL 6X Purple Loading Dye (NEB; Cat. B7024S) and 4 μL TAE buffer was added to the gel as a size standard.

### Multiplex sgRNA cassette expression plasmids

The multiplex plasmid pMMsgRNA (3TIIS) expression cassette, with an array of three separate sgRNA expression cassettes, was designed using the Extra Long sgRNA Array (ELSA) software (De Nova DNA; https://salislab.net/software/design_elsa_calculator) to contain three programable sgRNA cassettes (*Bbs*I, *Bsa*I, and *Bae*I spacer cloning sites) with non-homologous sequences. The insert was ordered as a synthetic dsDNA construct with 30 bp homologous ends (TWIST Bioscience, CA, USA) and cloned into a PCR amplified (primers pVSV105 cassette/MMsgRNA F/R (Table S2)) pVSV105 backbone using the NEBuilder® HiFi DNA Assembly Cloning Kit (NEB Cat. #E5520S) following the NEB protocol. The resulting plasmid, pMMsgRNA(3TIIS), is an empty cloning vector which can express three independent targeted sgRNAs from three different constitutive promoters. Cloning a 20-nt targeting spacer into a multiplexed sgRNA plasmid follows the same protocol described above for designing and cloning the spacer, and follows the same protocol for transforming the targeted multiplex vector into a *V. fischeri.* Strain with integrated *dcas9*. While the three sgRNA cassettes are not IPTG inducible, the *dcas9* is—so the multiplex CRISPRi system is inducible, but not independent. Four additional multiplex plasmid were constructed using a different protocol (—for these plasmids the synthetic dsDNA construct was designed to already contain 20-nt spacer sequences in the three sgRNA cassettes. All that was needed to construct these plasmids was to use HiFi Assembly (see above) and transform into *V. fischeri (see below).*

- pMMsgRNA(RR1)—targets site 1 in mRFP.
- pMMsgRNA(RR1:RR2)—targets sites 1 & 2 in mRFP.
- pMMsgRNA(RR1:LC1)—targets site 1 in mRFP and site 1 in *luxC.*
- pMMsgRNA(RR1:LC1:FA1)—targets site 1 in mRFP, site 1 in *luxC,* and site 1 in *flrA.*

### Dcas9 and dcas9/sgRNA plasmid *V. fischeri* strain construction.

Complete inducible CRISPRi *V. fischeri* strains were generated by introducing an inducible sgRNA expression plasmid, such as psgRNA (this study), from an *E. coli* cloning host by triparental conjugation with an *E.coli* thymidine- auxotroph containing conjugative helper plasmid pEVS104 (Stabb and Ruby 2002) and the appropriate *V. fischeri* recipient (ES114:pJMP1183 and ES114:pJMP1189) as described previously (DeLoney et al. 2002). Briefly, *E. coli* donor strains (i.e. psgRNA) and *E. coli* helper strain DH5α thy- carrying pEVS104 (Stabb and Ruby 2002) were cultured individually overnight in 5 mL LB media at 37 °C with appropriate antibiotics and additives. A recipient *V. fischeri* strain (e.g. ES114:pJMP1183; mFRP + , KanR) was cultured overnight (14 h) in SWT media at 28 °C with shaking. All cultures were then sub-cultured (100 μL aliquots into 5 mL appropriate media) and grown at their respective temperatures with shaking for 3–4 h. 250 μL of each of the three cultures were combined into a 1.5 mL microcentrifuge tube (Eppendorf). A 250 μL donor only control from each of the two *E. coli* cultures was placed in 1.5 mL microcentrifuge tubes, and a 250 μL recipient-only control was placed in a 1.5 mL microcentrifuge tube. The cells were pelleted at 8,000 rpm for 5 min in a microcentrifuge (Eppendorf), and the supernatant discarded leaving 10–20 μL liquid in the tube. The pellets were resuspended in this liquid, and 100 μL of each tube was spotted in the middle of a pre-warmed LBS agar plate. After the liquid had absorbed into the plates, they were inverted and incubated overnight in a 28 °C incubator. In the morning, the colony was scraped off with a sterile pipette tip and the cells resuspended in 1 mL LBS by vortexing briefly. 100 μL aliquots of each cell culture were spread onto LBS agar plates containing the appropriate antibiotic to select for plasmid transformed colonies (Chloramphenicol, 1–3 μg/μL). The plates are incubated at 18 °C to repress growth of donor *E. coli,* and colonies of plasmid bearing *V. fischeri* can be selected when they appear after 1–3 days. 20 of these colonies are isolated onto master LBS Kan 100 μg/mL agar plates and tested for the presence of the plasmid using plasmid extraction kits (Zymogen) and restriction digest analysis. Strains that have the sgRNA plasmid along with the chromosomally integrated dcas9 cassette will be CamR+, KanR+.

### Data analysis and graphing

GraphPad Prism Version 10.1 for Windows, GraphPad Software, (www.graphpad.com). One-way analysis of variance (ANOVA) was performed on multiple groups comparisons. Significance in the ANOVA analysis was followed by the use of between-group comparisons calculated using the Tukey’s Post Hoc comparison test. One-way or two-way ANOVAs and unpaired t-tests were used to analyze data for each graph as noted. For two-way ANOVA analyses, Šídák’s multiple comparisons test was used.

### *Euprymna scolopes* husbandry and hatchling inoculation protocols

*E. scolopes* husbandry and hatchling inoculation protocols are detailed in Supplementary File 1. The Nishiguchi laboratory’s squid husbandry room at UC Merced is IACUC certified for all related experiments.

### Luminescence measurement and quantification of symbiotic bacteria in LO

Luminescence produced by *V. fischeri* colonized hatchlings was measured with a Turner Designs TD20/20 luminometer (Turner Design). Juveniles were placed in 5 mL of sterile artificial seawater in 30 mL glass scintillation vials, and light produced was recorded for 15 s. Triplicate measurements (arbitrary light units, LU, per animal) were obtained and averaged.

The number of *V. fischeri* cells colonizing the squid light organ were assayed using LBS agar plates. Individual hatchlings to be assayed (typically after having their luminescence reading taken) were rinsed three times in 5 mL of sterilized artificial seawater. Juveniles were homogenized in a 1.5 mL Eppendorf microtubes using sterile 1.5 mL plastic pestles (Bel-Art) with 0.5 mL of sterile artificial seawater. 100 μL of the homogenate was used after serial dilution to plate in triplicate on LBS agar plates incubated at 28 °C. At 24 h colonies were counted to determine the colony forming units/light organ (CFU/LO).

### In vitro luminescence, fluorescence, and growth.

Strains were grown with shaking at 28 °C in SWT, with and without CRISPRi induction. At regular intervals, 1 mL aliquots of each culture were assayed for cell density (Optical density (OD_600_) via absorbance at 600 nm) with 1 mL cuvettes in a UV-3100PC spectrophotometer (VWR) and 5 mL aliquots in 30 mL glass scintillation vials were measured for luminescence (relative light units, RLU) in a Turner Designs TD-20/20 luminometer. A SpectraMax iD5 multimodal platereader was also used for measuring OD_600_, and for bioluminescence production. Fluorescence of mRFP tagged CRISPRi strains could also be quantified (ext: 590, emm: 612), and read in conjunction with OD_600_ and luminescence.

### Motility assays

For motility repression experiments, *V. fischeri* ES114 (control) and *V. fischeri* CRISPRi strains targeting *flrA* were grown overnight in TBS broth (1% tryptone, 2% sodium chloride) at 28 °C, with and without IPTG inducer supplementation as indicated, then sub-cultured in the same medium for 2 h at 28 °C (static). Cells to be assayed were normalized to an OD_600_ of 0.2 and 10 μL were drop spotted onto TBS soft agar plates with and without IPTG inducer supplementation as indicated, and incubated at 28 °C. Migration distance was measured hourly (as diameter to outer motility ring, mm) and plotted versus time.

## Supplementary Information

Below is the link to the electronic supplementary material.

Supplementary file1 (DOCX 202 KB)

## Data Availability

Data is provided within the manuscript or supplementary information files.
