# Supplementary material for: Generation and validation of a versatile inducible multiplex CRISPRi system to examine bacterial regulation in the Euprymna-Vibrio fischeri symbiosis
Source: Arch Microbiol. 2025 May 17;207(7):147. doi: 10.1007/s00203-025-04354-8 (PMC12085384; doi:10.1007/s00203-025-04354-8)
Supplement: Supplementary file 1 — Supplementary file1 (DOCX 202 KB) [file 203_2025_4354_MOESM1_ESM.docx]

**Supplementary File 1**

**Dcas9 expression toxicity testing**

The successful recovery of positive colonies of *V. fischeri* strains ES114:JMP1183 and ES114:pJMP1189 as well as *V. fischeri* strain ES114:JMP1183/psgRNA(RR1), which all contained an inducible *dcas9* cassette, demonstrated that basal levels of uninduced expression of dcas9 were not lethal to *V. fischeri*. However, as dcas9 has been reported to display cell toxicity at high expression levels in some cells (Rostain et al., 2023), we proceeded to test for dcas9 plus sgRNA expression toxicity at titrated levels of IPTG inducer supplementation. *V. fischeri* strains ES114:JMP1183, ES114:pJMP1189, ES114:JMP1183/psgRNA(*BsaI*), and ES114:JMP1183/psgRNA (RR1), were grown overnight (14 hour) in 5 mL SWT + Kan (100 μg/mL) media at 28°C with shaking (a ES114 WT control was grown in media with no Kan), and sub-cultured to a starting OD_600_ of 0.05 in SWT + Kan (100 μg/mL) media. Media was titrated with increasing amounts of IPTG to induce expression of dcas9 and sgRNA in plasmid constructs. 10 mL of cells were grown at 28°C with shaking, and 1 mL aliquots removed every hour for OD_600_ measurements until OD_600_ ~ 0.4 were reached. At IPTG levels of 0, 0.1, 0.5, 1.0, 1.5, and 2.0 mM there was no significant difference in growth of the four CRISPRi strains compared to WT ES114 (Fig. S1).


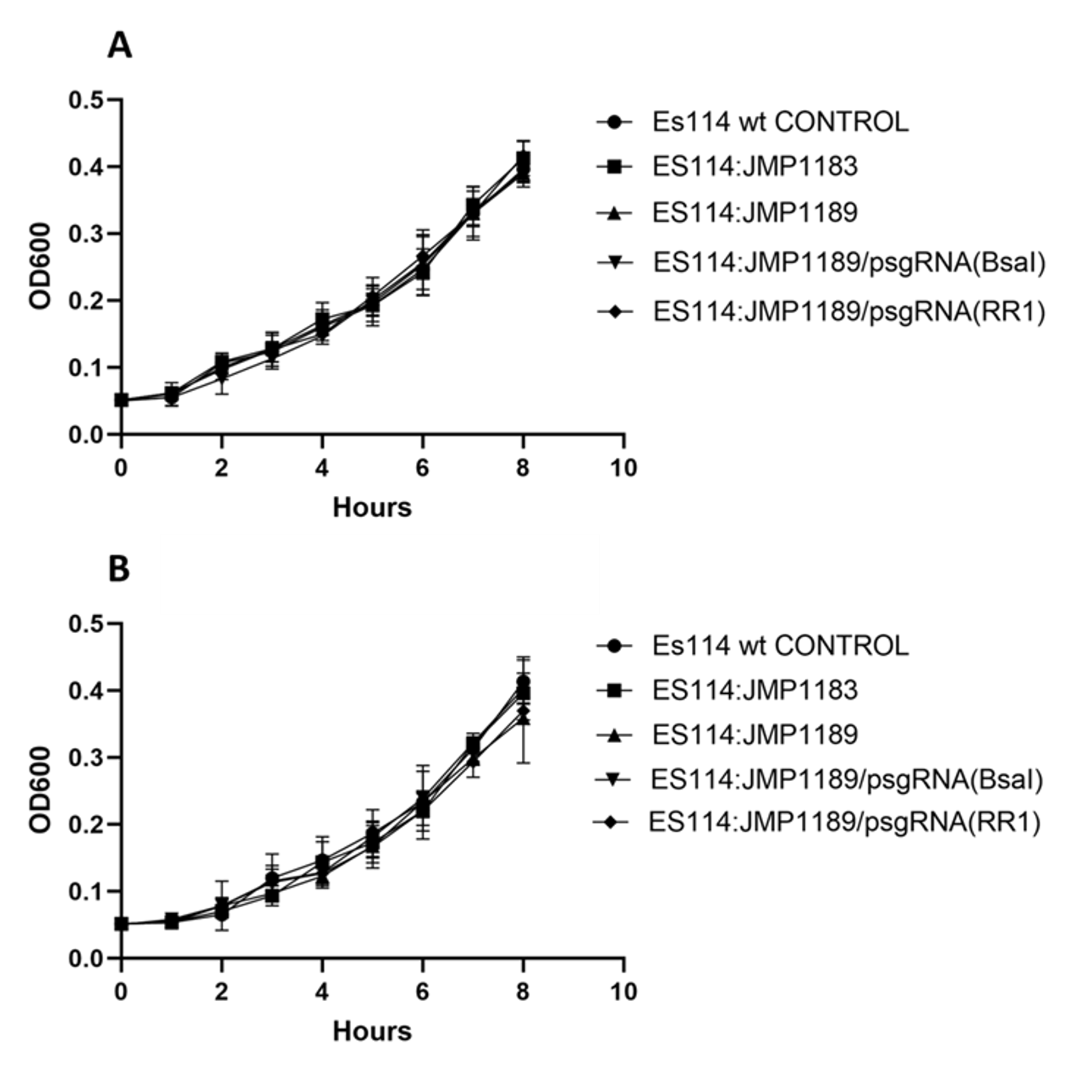


**Figure S1** Dcas9 toxicity testing. *V. fisheri* strains ES114 *wt*, ES114:JMP1183, ES114:pJMP1189, ES114:JMP1183/psgRNA (*BsaI*), and ES114:JMP1183/psgRNA (RR1) grown in media supplemented with A) 0 mM IPTG inducer, B) 2 mM IPTG inducer (intermediate levels not shown). Measurements were taken in triplicate, with each point representing the mean, and error bars the standard deviation.

***Euprymna scolopes* husbandry**

Adult *E. scolopes* were collected at Paiko Bay, O’ahu, Hawaii. Squids were maintained in running seawater aquaria at the University of Hawaii Kewalo Marine Laboratory until shipment. Squids were shipped to UC Merced and acclimated to the temperature and salinity (25°C, 34 ppt) of the aerated artificial seawater (Instant Ocean) circulating in our aquaria. Adult squids were kept in individual aquariums (50 x 30 X 60 cm) on a 12-hour day/night cycle and fed once per day with fresh-water ghost shrimp. Ammonia, nitrite, and nitrate levels were monitored twice weekly with a DR3900 spectrometer (Hach) using the Nitrogen-Ammonia Reagent Set (Hach). Water was changed when salinity rose above 36 ppt, or ammonia/nitrite/nitrate levels rose above 0.1/0.1/10 ppm respectively.

Mating of adult squids was conducted roughly every four days, with one male placed (via transfer in a small glass bowl) in the tank of one female and left over night. The male was returned to his tank the next morning; females generally produce eggs within 1-2 days after mating on the underside of PVC half-dome shelters. Eggs were collected, rinsed in sterile artificial seawater, rinsed with 1% bleach, and then rinsed twice with sterile artificial seawater to remove any *V. fischeri* from their surface. The rinsed eggs were placed in isolated juvenile aquariums and allowed to develop in the circulating artificial seawater kept at the same conditions as the adult tanks. Upon hatching (~25 days after being laid), the juvenile squid were collected within 3 hours for use in experiments.

**Colonization of hatchlings with *V. fischeri***

Hatchling *Euprymna scolopes* were collected within 4 hours of hatching. Hatchlings were removed with sterile plastic eye droppers and isolated in aerated artificial seawater at 23°C. Individual squids were placed in glass scintillation vials in 5 mL of artificial seawater containing ~ 5,000 colony forming units (CFU)/mL of WT *V. fischeri* ES114 (control), or one of the various CRISPRi *V. fischeri* strains. After 4 hours, individual squid were then washed twice in sterile artificial seawater and placed in their own scintillation vial filled with 5 mL of artificial seawater. Luminescence emission (relative light units; RLU) for each squid was measured at intervals up to 96-hrs post infection using a Turner Designs TD-20/20 luminometer. Luminescence emission was used to assess colonization success, and when appropriate, CRISPRi repression of bioluminescence. To assess squid colonization cell numbers, juvenile squids were sacrificed at time points up to 96-hrs post inoculation, homogenized (see below), and aliquots plated on appropriate selective media plates. All animal experiments were conducted in compliance with Institutional Animal Care and Use Committee protocols, University of California, Merced.

**Hatchling Inoculation Protocol**

*V. fischeri* cultures used to inoculate hatchlings were sub-cultured from 14-hour overnight cultures at 0.05 OD_600_ in the morning and allowed to grow at 28C with shaking to an OD_600_ ~0.3. All strains used were then diluted with their appropriate media into 50 mL of aerated artificial seawater at a concentration of 5 × 10^3^ CFU/mL. After the 12 noon room lighting switch to the 12-hour dark period, individual hatchlings were placed into 4 mL of this inoculation solution in a sterile 30 mL scintillation vial using sterile 1 mL plastic droppers. The vials were loosely covered with plastic wrap and allowed to sit for 4 hours. Then the inoculated hatchlings were washed twice by moving them with sterile 1 mL droppers into small glass bowls with 100 mL sterile aerated artificial seawater, letting them sit for 5 minutes, and moving them to a second small glass bowl with 100 mL of aerated sterile artificial seawater. Finally, they were transferred by sterile 1 mL droppers into new individual sterile scintillation vials with 5 mL of aerated artificial seawater. The inoculated hatchlings were kept in these vials under loose plastic film until 12 hours post inoculation when they were assayed for colonization using the proxy of luminescence production. To assay for luminescence, hatchlings in their individual scintillation vials were placed in a TD20/20 luminometer and luminescence was collected for 15 seconds to give a reading in relative light units. The assay was performed three times, and the highest reading recorded. Hatchlings that had not been inoculated but had gone through the same inoculation routine were used as controls for non-luminescence/colonization. Hatchlings were scored as successfully colonized if their luminescence reading at 12-hours post inoculation was at least 1000 RLU higher than that of the uncolonized controls (which are essentially non-luminescent). Successfully colonized hatchlings were returned to their vials, with their water being changed at every 12-hour light/dark period change. The infected squids followed this routine of monitoring their luminescence 12-hours later to give a 24-hour luminescence reading and were then monitored every 24-hours up till 96 hours post inoculation. The majority of hatchlings survived until at least the 72-hour time-point, but the colonization experiments were repeated an average of 6 times to collect enough data for analysis at later time points. At each 24-hour time point, three of the surviving colonized hatchlings were sacrificed via homogenization in 500 uL of sterile artificial seawater in a 2 mL Eppendorf tube. The homogenate (100 uL) was serially diluted 3x at 1:10 and spread on SWT agar plates and incubated at 28°C for 24 hours before colonies were counted.

**Table S1** Bacterial strains and plasmids used in this study.

| **Strain or plasmid** | **Description** | **Reference or source** |
| --- | --- | --- |
| **Bacterial strains** |  |  |
| ***V. fischeri*** |  |  |
| ES114 | Wild type isolate from *Euprymna scolopes* light organ | (Boettcher & Ruby, 1990) |
| ES114:pJMP1183 | ES114 Tn7:: (*yeiR-dcas9-Kan-mRFP-sgRNA(mRFP)/glmS*) | This study |
| ES114:pJMP1189 | ES114 Tn7:: (*yeiR-dcas9-Kan-mRFP/glmS*) | This study |
| ES114:CRISPRi/NT | ES114:pJMP1189 carrying psgRNA(NT) |  |
| ES114:CRISPRi/mRFP | ES114:pJMP1189 carrying plasmid psgRNA(RR1) | This study |
| ES114:CRISPRi/luxC | ES114:pJMP1189 carrying plasmid psgRNA(LC1) | This study |
|  |  |  |
| ES114/MCRISPRi(RR1) | ES114:pJMP1189 carrying plasmid pMMsgRNA(RR1) | This study |
| ES114/MCRISPRi(RR1,RR2) | ES114:pJMP1189 carrying plasmid  pMMsgRNA(RR1:RR2) | This study |
| ES114/MCRISPRi(RR1,LC1) | ES114:pJMP1189 carrying plasmid  pMMsgRNA(RR1:LC1) | This study |
| ES114/MCRISPRi(RR1:LC1:FA1) | ES114:pJMP1189 carrying plasmid  pMMsgRNA(RR1:LC1:FA1) | This study |
| ***E. coli*** |  |  |
| DH5α | *fhuA2Δ(argF-lacZ)U169 phoA glnV44 Φ80Δ(lacZ)M15 gyrA96 recA1 relA1 endA1 thi-1 hsdR17* | NEB |
| GT115 | *F- mcrA ∆(mrr-hsdRMS-mcrBC) φ80lacZ∆M15 ∆lacX74 nupG recA1 araD139 Δ(ara-leu)7697 galE15 galK16 rpsL(StrA) endA1 ∆dcm uidA(∆MluI)::pir-116 ∆sbcC-sbcD* | Invivogen |
| CC118 λpir /pEVS104 thy- | *A(ara-leu) araD AlacX74 galE galK phoA20 thi-J rpsE rpoB argE(Am) recAl pir* | (Herrero et al., 1990) |
| BW25141/pJMP1039 | *Δ(araD-araB)567 ΔlacZ4787(::rrnB-3) Δ(phoB-phoR)580 λ- galU95 ΔuidA3::pir+ recA1 endA9(del-ins)::FRT rph-1 Δ(rhaD-rhaB)568 hsdR514* | Peters et al., 2019) |
| **Plasmids** |  |  |
| pESV104 | Conjugal Helper plasmid (*tra+, mob+*, kn^r^) | (Eric V. Stabb and Ruby 2002) |
| pVSV105 | Shuttle vector (cm^r^) | (Dunn et al. 2006) |
| pJMP1183  (Addgene: 119254) | Mobile-CRISPRi Tn7 transposon: KanR, dcas9, mRFP, sgRNA(mRFP) | Addgene |
| pJMP1189  (Addgene:119257) | Mobile-CRISPRi Tn7 transposon: KanR, dcas9, mRFP | Addgene |
| pJMP1039  (Addgene:119239) | Mobile-CRISPRi Tn7 transposase, AmpR | Addgene |
| psgRNA(BsaI) | Empty sgRNA for cloning, cmr, Vf *oriV*, *oriT*, R6Kg *oriV* | This study |
| psgRNA(NT) | sgRNA Non-Targeting, *cmr*, Vf *oriV,* *oriT*, R6Kg *oriV* | This study |
| psgRNA(RR1) | sgRNA targeting mRFP site 1, *cmr*, Vf *oriV,* *oriT*, R6Kg *oriV* | This study |
| psgRNA(LC1) | sgRNA targeting *luxC* site 1, *cmr,* Vf *oriV, oriT*, R6Kg *oriV* | This study |
| pMMsgRNA(3TIIS) | Multiplex sgRNA with three empty cloning sites, *cmr*, Vf *oriV, oriT*, R6Kg *oriV* | This study |
| pMMsgRNA(RR1) | Multiplex sgRNA with one mRFP spacer, *cmr*, Vf *oriV, oriT*, R6Kg *oriV* | This study |
| pMMsgRNA(RR1:RR2) | Multiplex sgRNA with two mRFP spacers, *cmr*, Vf *oriV, oriT*, R6Kg *oriV* | This study |
| pMMsgRNA(RR1:LC1) | Multiplex sgRNA with one mRFP spacer and one luxC spacer, *cmr*, Vf *oriV, oriT*, R6Kg *oriV* | This study |
| pMMsgRNA(RR1:LC1:FA1) | Multiplex sgRNA with one mRFP spacer, one *luxC* spacer, and one *flrA* spacer, *cmr*, Vf *oriV, oriT*, R6Kg *oriV* | This study |

**Table S2** Oligos and synthetic dsDNA constructs used in this study

| **Name** | **Sequence** | **Purpose** |
| --- | --- | --- |
| **Oligos** |  |  |
| sgRNA(*Bsa1*) F: | tctaatgtgcgaacaagtctgcagacgtaaaaaaagcggcgtggt | sgRNA/lacI(*BsaI*) cassette |
| sgRNA(*BsaI*) R: | ttcgcgtcagcgggtcttcttgagaagagaaaagaaaaccgccgatcctgtccac | sgRNA/lacI(*BsaI*) cassette |
| pVSVb1 F: | tctcttctcaagaagacccgctgacgcgaaccc | pVSV105 backbone |
| pVSVb1 R: | ttacgtctgcagacttgttcgcacattagaaaagaaagtgggcttaactcgct | pVSV105 backbone |
| VfsgRNAcon F: | ttaaataaggctagtccgttatcaacttgaaaaagtggcac | Cloning conf. |
| VfsgRNAcon R: | attatggtgaaagttggaacctcttacgtgcc | Cloning conf. |
| NT F: | tagtgggccctaaaaccccctttt | Non-targeting spacer |
| NT R: | aaacaaaagggggttttagggccc | Non-targeting spacer |
| RR1 F: | tagtaactttcagtttagcggtct | mRFP spacer |
| RR1 R: | aaacagaccgctaaactgaaagtt | mRFP spacer |
| RR1con F: | aaacagaccgctaaactgaaagttactagaagtatcttgt | Cloning conf. |
| RR1con R: | attatggtgaaagttggaacctcttacgtgcc | Cloning conf. |
| LC1 F: | tagtatcgttgacctacggtatat | luxC spacer |
| LC2 R: | aaacatataccgtaggtcaacgat | luxC spacer |
| LC1con F: | gtgcgatcgggctagggct | Cloning conf. |
| LC1con R: | ttaggctagagatatcgg | Cloning conf. |
| pVSV105 cassette/MMsgRNA F: | acccgctgacgcgaac | pVSV105 backbone for MMsgRNAs |
| pVSV105 cassette/MMsgRNA R: | tgttcgcacattagaaaagaaagtggg | pVSV105 backbone for MMsgRNAs |
| **dsDNA constructs** |  |  |
| MMsgRNA  (3TIIS) | aagcccactttcttttctaatgtgcgaacattgacagtagatcagagggttgctataatcgacagtgagaccaactttggtctccaccatagcggtcggtctctgttgtagatctagaaatagaatgttacaattaggctagtccgttatgaacatgaaaatgtgagaaaagaggccgcgaaagcggccttttttcgttttcacttctctgttggcacgaaaagggcaataagatttacggattactatcttgacactaccgagacagtgacatataataggaccgcgtcttcaagatcgaagactagatttcgagctaggcatagcaagtgaaattaaggctggtccattaacaccttgaaaaagggaacaataaggcctccctttagggggggccttttttattgatgcggataaagttgatacccttacctgagttcttctgaaaataacggactttgacacgatgcttgctgctacctataataacatacatgcgtaggacttacgtaccggtagatgatgtagatgtagaaatacaaggttacattaaggcccgtccgtaatcaacttgaagaagtgttccatcgggtccgaattttcggaccttttctccgcatgaaaagcaatccctcgtgaacccgctgacgcgaaccccttgcggccgca | This study |
| MMsgRNA  (RR1) | aagcccactttcttttctaatgtgcgaacattgacagtagatcagagggttgctataatcgacagaactttcagtttagcggtctgttgtagatctagaaatagaatgttacaattaggctagtccgttatgaacatgaaaatgtgagaaaagaggccgcgaaagcggccttttttcgttttcacttctctgttggcacgaaaagggcaataagatttacggattactatcttgacactaccgagacagtgacatataataggaccgcgtcttcaagatcgaagactagatttcgagctaggcatagcaagtgaaattaaggctggtccattaacaccttgaaaaagggaacaataaggcctccctttagggggggccttttttattgatgcggataaagttgatacccttacctgagttcttctgaaaataacggactttgacacgatgcttgctgctacctataataacatacatgcgtaggacttacgtaccggtagatgatgtagatgtagaaatacaaggttacattaaggcccgtccgtaatcaacttgaagaagtgttccatcgggtccgaattttcggaccttttctccgcatgaaaagcaatccctcgtgaacccgctgacgcgaaccccttgcggccgca | This study |
| MMsgRNA  (RR1:RR2) | aagcccactttcttttctaatgtgcgaacattgacagtagatcagagggttgctataatcgacagaactttcagtttagcggtctgttgtagatctagaaatagaatgttacaattaggctagtccgttatgaacatgaaaatgtgagaaaagaggccgcgaaagcggccttttttcgttttcacttctctgttggcacgaaaagggcaataagatttacggattactatcttgacactaccgagacagtgacatataataggaccgaccgacaggatgtcccaagcgaagatttcgagctaggcatagcaagtgaaattaaggctggtccattaacaccttgaaaaagggaacaataaggcctccctttagggggggccttttttattgatgcggataaagttgatacccttacctgagttcttctgaaaataacggactttgacacgatgcttgctgctacctataataacatacatgcgtaggacttacgtaccggtagatgatgtagatgtagaaatacaaggttacattaaggcccgtccgtaatcaacttgaagaagtgttccatcgggtccgaattttcggaccttttctccgcatgaaaagcaatccctcgtgaacccgctgacgcgaaccccttgcggccgca | This study |
| MMsgRNA  (RR1:LC1:FA1) | aagcccactttcttttctaatgtgcgaacattgacagtagatcagagggttgctataatcgacagaactttcagtttagcggtctgttgtagatctagaaatagaatgttacaattaggctagtccgttatgaacatgaaaatgtgagaaaagaggccgcgaaagcggccttttttcgttttcacttctctgttggcacgaaaagggcaataagatttacggattactatcttgacactaccgagacagtgacatataataggaccatcgttgacctacggtatatgatttcgagctaggcatagcaagtgaaattaaggctggtccattaacaccttgaaaaagggaacaataaggcctccctttagggggggccttttttattgatgcggataaagttgatacccttacctgagttcttctgaaaataacggactttgacacgatgcttgctgctacctataataacataggatcaccttaagattatgagatgtagatgtagaaatacaaggttacattaaggcccgtccgtaatcaacttgaagaagtgttccatcgggtccgaattttcggaccttttctccgcatgaaaagcaatccctcgtgaacccgctgacgcgaaccccttgcggccgca | This study |

**Table S3. Abbreviations used in text.**

| AHL | Acyl homoserine lactonePTG |
| --- | --- |
| CRISPRi | Clustered regularly interspaced short palindromic repeats, interfering |
| dcas9 | deactivated crispr associated protein 9 |
| IPTG | isopropyl-β-d-thiogalactopyranoside |
| mRFP | monomeric red fluorescent protein |
| sgRNA | single guide RNA |
| Tn7 | Transposon 7 |
